# Total Hip Arthroplasty—Current Challenges

**DOI:** 10.3390/medicina59061011

**Published:** 2023-05-24

**Authors:** Johannes Dominik Bastian

**Affiliations:** Department of Orthopaedic Surgery and Traumatology, Inselspital, Bern University Hospital, University of Bern, 3010 Bern, Switzerland; johannes.bastian@insel.ch; Tel.: +41-31-6322111

In 1891, Professor Themistocles Glück in Germany was the first to replace a femoral head in hip joints destroyed by tuberculosis using ivory [1], followed by Marius Smith-Petersen in 1925 using a mold arthroplasty made out of glass [2]. Further attempts in hip joint replacement followed, until Sir Jon Charnely significantly advanced total hip arthroplasty in the 1960s [3]. In the following decades, further innovations in materials and design enhanced the outcome. Because of the enhanced survivorship and low revision rates in 2007, total hip arthroplasty (THA) was called the “operation of the century” [4]. To date, hip surgeons have to face various challenges currently and in the future in the fields of primary and revision surgeries, primary complex THA, and complication management, in specific patients and must identify optimal patient management protocols and the value of new technologies in cutting-edge research topics to improve the overall outcomes and simplify interventions.

This Special Issue of *Medicina*, entitled “Total Hip Arthroplasty—Current Challenges”, includes 11 articles that deal with patient selection and preparation for THA, implant selection, the impact of surgical techniques on implant positioning or biomechanics, and complication management.

Total hip arthroplasty is an elective and irreversible procedure. Lützner et al. [5] report the recommendations from a German consensus initiative to identify patients for whom the benefit of a THA may exceed the potential risks. The so-called “Evidence- and consensus-based indication criteria for total hip replacement (EKIT-Hip)” initiative developed a clinical practice guideline with an accompanying algorithm to guide consultations on THA. Decision making on THA relies on either minimum requirements, such as the confirmation of diagnosis, patients’ individual burden of illness, alternative treatment options and contraindications, and further considerations, such as modifiable risk factors and shared decision making. Adherence to the presented algorithm enhances the standardization of consultations, the quality of health care and patient satisfaction. 

In their current concepts review, Solarino et al. [6] questioned the sex and gender-related differences in the outcome of patients undergoing THA in order to improve clinical outcomes and prevent post-operative complications. The results are reported in relation to the surgical approach, robotic surgery, scar cosmesis, implant choice, post-operative clinical outcome, complications, and sexual activity after THA, in addition to psychological status and daily functional requirements. In conclusion, female patients need more specific attention to improve outcomes, reduce complication risks and manage patient satisfaction. Accordingly, the principles of a gender-specific approach should be applied in daily clinical practice and planning. 

Once patients are selected for THA, knowledge about the effects of prehabilitation on the post-operative outcomes is of value. Widmer et al. [7] systematically reviewed the literature and included 14 studies out of 400 potentially relevant studies on whether prehabilitation before THA in the form of exercise therapy, education alone or both improves physical functioning compared to no intervention. Exercise was an effective prehabilitation measure with regard to post-operative physical functioning, whereas education alone did not offer any additional benefit over usual care. 

Dislocation is one of the reasons for revision surgery. Aguado-Maestro et al. [8] reported their results in a study for the routine choice of dual mobility cups (DMC) in THA patients. In the analysis, 531 patients who underwent arthroplasties (mean age 72 years) with a mean follow-up of 2.8 years were included and assessed for the treatment received, new dislocations, and need for surgical revision. During the study period (2015 to 2021), the trend of indications for DMC increased from 16% to 78%. Dislocation was associated with smaller heads (22 mm), cups and a posterior approach. None of the dislocations were associated with elective surgery. Hanslmeier et al. [9] reported their results of a retrospective study with a 6-year follow-up with femoral head and liner exchange in patients with dislocations unrelated to trauma, component malpositioning or component loosening. The Kaplan–Meier analysis revealed a revision-free implant survival rate from any cause of 80% (confidence interval 95%: 64.3–99.6%) at 5 years after the head and liner exchange (index surgery).

Tanaka et al. [10] analyzed the impact of the hip center position on anterior-based muscle-sparing (ABMS) total hip arthroplasty for post-operative hip muscle strength. In their retrospective cohort study, in 38 patients, muscle strength was assessed using isokinetic dynamometry before the operation, 6 and 12 months after surgery. The abductor muscle strength was significantly decreased at 6 months in presence of a vertical shift of the center of rotation of more than 15 mm. The impact of various techniques used to restore native hip mechanics and correct the placement of the acetabular component was reported by a further three articles [11,12,13]. Zurmühle et al. [11] assessed in a retrospective, accuracy study in a single surgeon case series of 367 image-less cup-navigated, primary THAs performed through an anterolateral approach for individual adjustments, accuracy, precision and robustness. This image-less navigation achieved adequate placement of the acetabular components with a minimum number of outliers and offers an additional tool to address challenging hip prosthesis in the context of the hip–spine relationship. Brady et al. [12] report the accuracy and reliability of software navigation for acetabular component placement in THA. Their data validate a novel method for calculating the 3D orientation of the acetabular cup from 2D fluoroscopic imaging with an excellent interrater and intrarater reliability and excellent intermethod accuracy. In the review by Ogilvie et al. [13], the workflow, outcomes and the role of addressing the challenge of spinopelvic imbalance of robotic-arm-assisted (Ro) THA are described and illustrated by a case presentation. The authors emphasize that robotic technology supports the concept of personalized component positioning based on the patients’ phenotypes, resulting in improved radiological outcomes and has the potential to tackle the challenges posed by abnormal motion in the spinopelvic kinetic chain. The cost effectiveness of RoTHA is still a concern and the evaluation of any functional benefits needs to be assessed in detail in prospective randomized controlled trials with longer-term follow ups. 

Finally, this Special Issue includes two articles that report on acetabular peri-prosthetic fractures [14,15]. Literature on this subject is sparse, no universally recognized treatment algorithm exists and surgical management represents a challenge for most orthopedic surgeons. Beckers et al. [14] present in their narrative review an update on epidemiology, diagnostic work-up, the existing classification systems, surgical approaches and therapeutic options. Their treatment algorithm provides an individualized therapeutic strategy in this rare but challenging complication after THA. In a retrospective case series study, Ivanova et al. [15] report on the factors that contribute to medical cup protrusion following excessive reaming in primary THA in older adults. Revision surgery of these failed primary THAs following medial wall perforation was successful using a Ganz reinforcement ring combined with bone grafts and plating of the posterior column and/or screws for the anterior column. Surgeons should be aware of this rare and probably underreported complication and restore the anatomic center of rotation by intraoperatively treating the defect.

Overall, the articles published in this Special Issue contribute to a deeper understanding of the current challenges in THA, raising awareness, and promoting further research in the field.

## Data Availability

Not applicable.
